# Investigation of the active compounds and pharmacological mechanisms of *Qufengxuanfei decoction* in the treatment of chronic cough

**DOI:** 10.3389/fmed.2026.1748916

**Published:** 2026-04-30

**Authors:** Zhilin Liu, Jiashan Li, Kun Ji, Shaohui Wen, Shangjuan Dong, Ying Wang, Jingyue Luo, Jianling Ma, Liqing Shi

**Affiliations:** 1 The First Affiliated Hospital of Guangzhou Medical University, Guangzhou, Guangdong, China; 2 The First Affiliated Hospital of Guangzhou Medical University, Hengqin Hospital / Hengqin Guangdong-Macao In-Depth Cooperation Zone Central Hospital, Hengqin, Guangdong, China; 3 Second School of Clinical Medicine, Beijing University of Chinese Medicine, Beijing, China; 4 Institute of Acupuncture and Moxibustion, China Academy of Chinese Medical Sciences, Beijing, China; 5 Department of Respiration, Dongfang Hospital, Beijing University of Chinese Medicine, Beijing, China; 6 Department of Respiration, Zhejiang Provincial Hospital of Traditional Chinese Medicine, Hangzhou, Zhejiang, China; 7 Department of Traditional Chinese Medicine, Beijing Chest Hospital, Capital Medical University, Beijing, China

**Keywords:** airway neurogenic inflammation, animal experiment, chronic cough, molecular docking, network pharmacology, Qufengxuanfei decoction

## Abstract

**Objective:**

To investigate the mechanism of the *Qufengxuanfei Decoction* (QFXFD) in improving airway neurogenic inflammation in chronic cough through network pharmacology and animal experiments.

**Methods:**

The active components and potential targets of QFXFD were identified using the Traditional Chinese Medicine Systems Pharmacology Database and Analysis Platform (TCMSP). Disease-related targets associated with chronic cough were retrieved from the GeneCards, OMIM, and TTD. The intersection of drug targets and disease targets was determined, and a “TCM–bioactive compounds–intersection targets” network was constructed using Cytoscape software. Protein–protein interaction (PPI) analysis of the intersecting targets was performed via the STRING database to identify core genes. Gene Ontology (GO) and Kyoto Encyclopedia of Genes and Genomes (KEGG) enrichment analyses were subsequently conducted. Molecular docking was performed using AutoDock and related tools to validate the interactions between key active compounds and core targets. A guinea pig model of heightened cough sensitivity was established, followed by intervention with QFXFD. Cough frequency, differential cell counts in bronchoalveolar lavage fluid (BALF), and histopathological changes in lung tissue were evaluated. In addition, the expression levels of PLC-*β*, PKC, TRPA1, TRPV1, and SP/NK-1R in lung tissues were assessed.

**Results:**

The key bioactive constituents of QFXFD responsible for alleviating airway neurogenic inflammation in chronic cough were identified as quercetin, *β*-sitosterol, luteolin, kaempferol, stigmasterol, and tanshinone IIA. A total of 207 intersecting targets between QFXFD and chronic cough were identified, with core targets including TP53, AKT1, IL6, MAPK1, MAPK3, TNF, BCL2, CASP3, ESR1, CCND1, HSP90AA1, and MYC. These shared targets were significantly enriched in 838 biological processe (BP), 188 molecular function (MF), 104 cellular component (CC), and 179 KEGG pathways. Molecular docking analyses demonstrated favorable binding affinities between tanshinone IIA and TP53, luteolin and TP53, kaempferol and TNF, as well as quercetin and TNF. Animal experiments showed that the QFXFD could significantly improve the peripheral cough hypersensitivity state of guinea pigs with increased cough sensitivity, reduce the frequency of cough, decrease the proportion of neutrophils and eosinophils in BALF, improve the pathological damage of lung tissue, and also reduce the expression levels of PLC-*β*, PKC, TRPA1, TRPV1, and SP/NK-1R.

**Conclusion:**

QFXFD exerts its therapeutic effects through a multi-component, multi-target, and multi-pathway mechanism. Its diverse active constituents modulate a variety of signaling pathways and regulate key biological processes related to inflammation and immune responses, thereby contributing to the improvement of airway neurogenic inflammation and airway hyperresponsiveness. These findings provide a valuable theoretical and experimental basis for the clinical application and further development of QFXFD as a potential therapeutic strategy for chronic cough.

## 1 Introduction

Chronic cough is defined as a condition characterized by cough as the primary or sole symptom lasting longer than 8 weeks, with no significant abnormalities detected on X-ray chest films (1). The global prevalence of chronic cough is approximately 10% (2), although variations exist among different countries and regions due to environmental and genetic factors. Data from the US National Health and Nutrition Examination Survey indicate a prevalence rate of 5% (3), whereas an epidemiological study in Northern Europe reported a prevalence of 16% (4). In China, the reported prevalence ranges from 2.0 to 28.3% (5–9). According to a study by Professor Lai Kefang’s team, based on patients from specialized cough clinics, the gender distribution among chronic cough patients in China is nearly equal (51.5% vs. 48.5%), with the highest incidence observed in the 30–39 age group (10). Overall, chronic cough has a high prevalence, complex etiology, and is often subject to misdiagnosis and inappropriate treatment. Patients frequently undergo repeated diagnostic procedures or prolonged use of antibiotics and antitussive medications, often with limited therapeutic benefit and significant adverse effects. This condition severely affects patients’ work, quality of life, and mental health. As such, chronic cough has garnered increasing attention from clinicians and has become a major challenge and focal point in respiratory research. Consequently, the search for effective and targeted therapeutic strategies is of critical importance.

Chronic cough falls under the category of “persistent cough” or “stubborn cough” in Traditional Chinese Medicine (TCM). TCM has a long-standing history in the understanding and treatment of cough, with numerous classical physicians having contributed extensive and valuable therapeutic experience over centuries. The TCM approach to managing chronic cough is rich in clinical practice, with well-documented efficacy and minimal side effects. Consequently, it has garnered increasing attention, and many clinical cases have shown significant improvement following TCM treatment. Since 2015, the *Guidelines for the Diagnosis and Treatment of Cough* have also incorporated TCM-based therapies. Chronic cough is characterized by symptoms such as throat itching that triggers coughing, cough induced by unusual odors, and sudden, paroxysmal onset and cessation of symptoms. These features align with TCM theory, which holds that “wind tends to move and change swiftly, causes spasms, and without wind, there is no itch,” suggesting that *wind evil* (*feng xie*) is a key pathogenic factor in chronic cough (11, 12). As noted in *Zá Bìng Yuán Liú Xī Zhú Gǎn Mào Yuán Liú* (The Origins and Development of Miscellaneous Diseases The Origins of Common Cold), “when wind evil invades the body, regardless of the entry point, it ultimately settles in the lung.” Furthermore, our retrospective analysis of 618 cases of chronic cough and an additional study on the TCM pattern distribution among 771 patients with chronic cough demonstrated the prominent role of *wind evil* in the disease onset (13, 14). These findings led to the conclusion that “wind pathogen invading the lung” is a fundamental pathogenesis of chronic cough in TCM. Based on this understanding, Professor Shi Liqing developed the *Qufengxuanfei Decoction* (QFXFD), a six-herb decoction designed to dispel wind evil, disseminate Lung Qi, and relieve cough. Previous studies conducted by our research group have confirmed the formula’s favorable clinical efficacy (15, 16).

This study aims to explore the underlying mechanisms of the QFXFD in the treatment of chronic cough through network pharmacology and molecular docking analyses, and to evaluate its effects on cough sensitivity and the level of airway neurogenic inflammation in guinea pigs with chronic cough. The findings are expected to provide a valuable theoretical foundation for the clinical application of the QFXFD.

## 2 Materials and methods

### 2.1 Pharmacodynamic evaluation of QFXFD

#### 2.1.1 Preparation of QFXFD

The QFXFD is composed of the following herbal ingredients: *Mahuang* (6 g), *Qingfengteng* (14 g), *Ziwan* (12 g), *Baibu* (10 g), *Qianhu* (10 g), and *Houpo* (6 g). All herbs were sourced from the Traditional Chinese Medicine Pharmacy of Dongfang Hospital, Beijing University of Chinese Medicine. The decoction was prepared by the hospital’s Pharmaceutical Preparation Center, and subsequently concentrated to a final crude drug concentration of 2 g/mL. The prepared decoction was stored at −80 °C for later use. To ensure the quality and consistency of the herbal preparation, the chemical profile of QFXFD was characterized using UPLC-Q-TOF-MS. A total of 10 major bioactive markers were identified. Detailed experimental parameters and results are provided in the Supplementary files.

#### 2.1.2 Animal model and drug administration

Sixty male Hartley guinea pigs (specific pathogen-free, SPF), aged 2 weeks and weighing 200–250 g, were purchased from Weitong Lihua Laboratory Animal Technology Co., Ltd. (Beijing, China). All experimental procedures were approved by the Animal Ethics Committee of Dongfang Hospital, Beijing University of Chinese Medicine (Approval No.: 201907) and were conducted in strict accordance with institutional guidelines and national regulations for the care and use of laboratory animals.

All guinea pigs were housed individually under controlled environmental conditions, with a temperature maintained at 23 °C and relative humidity between 50 and 60%. Animals had ad libitum access to food and water. After a one-week acclimatization period, the guinea pigs were randomly assigned into six groups (*n* = 10 per group): a normal control group, a model group (cough hypersensitivity model), a PLC inhibitor group, a TRPA1 inhibitor group, a TRPV1 inhibitor group, and a QFXFD group. Cough hypersensitivity was induced in all groups except the normal control group using the following protocol (17): Each guinea pig received an intraperitoneal injection of cyclophosphamide (30 mg/kg). Two days later, sensitization was initiated with an intraperitoneal injection of 1.5 mL of a suspension containing 2 mg of ovalbumin (OVA) and 100 mg of aluminum hydroxide. After 3 weeks, an additional intraperitoneal injection of the same suspension was administered to reinforce sensitization. Subsequently, the sensitized animals were exposed to aerosolized OVA (10 mg/mL) in a custom-built nebulization chamber for 60 s to induce cough. A cough was defined as a sequence of characteristic behaviors, including visible abdominal contractions accompanied by leg extension, neck stretching, mouth opening, and an audible cough sound. The number of coughs was recorded, and a model was considered successfully established if the animal exhibited more than 10 cough episodes within a 2-min observation period.

Beginning on the first day following successful model establishment, both the normal control and model groups received intragastric administration of normal saline. The PLC inhibitor group underwent daily aerosolized treatment with U73122 (1 mmol/L) for 5 min. Similarly, the TRPA1 inhibitor group received aerosolized HC-030031 (1 mmol/L) for 5 min per day. The QFXFD group was administered QFXFD intragastrically at a dose of 5 g/kg/day.

#### 2.1.3 Measurement of cough reflex sensitivity

After 7 consecutive days of intervention, challenge experiments were conducted on days 2 and 3. Each guinea pig was placed in a nebulization chamber and exposed to aerosolized capsaicin (50 μmol/L) and AITC (40 mmol/L) solutions for 5 min, respectively. During each challenge, a designated observer recorded the number of coughs. The total number of coughs within a 10-min observation period was recorded for each animal. Following the experiments, the guinea pigs were euthanized. Bronchoalveolar lavage fluid (BALF) was collected, and lung tissue samples were harvested for further analysis.

#### 2.1.4 Leukocyte classification and count in BALF

The guinea pigs were sacrificed by blood collection from the abdominal aorta. The chest was opened to expose the trachea, heart and lungs of the guinea pigs, and the right hilum of the lung was clamped with a hemostat. A syringe was used to aspirate 3 mL of 0.9% NS, and the connector was connected to the tracheal intubation interface. The lavage was repeated 3 times, and about 1.2 mL of BALF was recovered from each guinea pig. The sample was centrifuged at 3000 r/min for 10 min at 4 °C, and the supernatant was collected. The supernatant of BALF was taken, smeared on a slide and air-dried naturally, then stained with hematoxylin–eosin (HE). Leukocyte classification and count were performed under a light microscope.

#### 2.1.5 HE staining of lung tissue

Lung tissues were fixed in 4% paraformaldehyde, dehydrated, and embedded in paraffin. HE staining was subsequently performed to evaluate histopathological alterations and structural damage in the lung tissues.

#### 2.1.6 Western blot

Lung tissues were homogenized in lysis buffer using a tissue grinder, and the homogenates were centrifuged to collect the supernatant. Total protein concentration was determined using the BCA protein assay kit. Protein expression levels were quantified by calculating the ratio of the grayscale intensity of the target protein band to that of the internal loading control.

#### 2.1.7 RT-qPCR

Total RNA was extracted using TRIzol reagent and reverse-transcribed into cDNA. RT-qPCR was performed to assess gene expression levels, with the primer sequences listed in Table 1. Calculate the relative expression of mRNA according to the formula RQ = 2^–(△Ctq–△Ctcb).

**Table 1:** Sequence of primers.

Gene	Forward 5′-3′	Reverse 5′-3′
SP	CCTGTTTGTGCTCCTACTGC	CTGTGCAGTTTCCCTTGCTT
PLC-β	AGGCAAGAGAAACTCGTGGA	AGTTCCATTTGCAGCTTGGG
PKC	ATGGAAATTGTGTTCGTTTATGCAT	CCTGGGGTCATCAGTGTCAGGTCCC
TRPA1	AGCCAGTTATGGGCGTATCA	AGCCATTGTGGTCACTGAGA
TRPV1	GAGAGATCCCGGAACCAGAG	AGCACCGAGTTCTTCTCACA

### 2.2 Network pharmacology study

#### 2.2.1 Screening of bioactive components and targets of QFXFD

The Traditional Chinese Medicine Systems Pharmacology Database and Analysis Platform (TCMSP)^1^ (18) was used to retrieve the bioactive components and protein targets of QFXFD. Criteria for inclusion were an oral bioavailability (OB) greater than 30% and a drug-likeness (DL) higher than 0.18, as recommended by the literature and TCMSP guidelines (19, 20). Specifically, OB represents the percentage of an orally administered dose that reaches the systemic circulation, while DL provides a qualitative assessment of a compound’s pharmaceutical potential based on its structural and physicochemical properties. These parameters are critical for filtering components with favorable pharmacokinetic profiles, ensuring that the selected candidates possess the capacity to exert biological effects *in vivo*. The resulting targets were then normalized using “*Homo sapiens*” in the Uniport database. Using Cytoscape 3.10.0, a network about QFXFD was established, and then using the network analysis tool in Cytoscape, core ingredients were obtained based on their degree values (21).

#### 2.2.2 Identifying targets associated with QFXFD intervention in chronic cough

Using “chronic cough” as a keyword, we searched for potential targets for chronic cough in the GeneCards,^2^ OMIM^3^ and TTD^4^ databases. Then we used the Venn diagram to identify common targets between the bioactive components of QFXFD and the primary relevant targets of chronic cough, with the intersecting portion being considered as potential therapeutic targets for QFXFD intervention in chronic cough.

#### 2.2.3 Analyzing the network of intersecting targets

A protein–protein interaction (PPI) network of intersecting targets was constructed and visualized using the STRING 12.0 database and Cytoscape 3.10.0 (22) and then the network topological properties (degree, betweenness, and closeness) were evaluated. Further explorations were carried out using CtyoNCA.

#### 2.2.4 Gene ontology and Kyoto encyclopedia of genes and genomes analyses

These targets were subjected to GO and KEGG analysis using the DAVID^5^ database. Three GO characteristics were analyzed: biological process (BP), cellular component (CC), and molecular function (MF). Classification and summary of the top 30 KEGG pathways. The enrichment results were visualized through the Bioinformatics Analysis Platform.^6^

#### 2.2.5 Molecular docking

The 3D structure of the target proteins and the 2D structure of the active ingredients were downloaded from the PDB^7^ database and the PubChem^8^ database. The proteins were then processed using PyMOL software to remove water molecules, phosphate groups, and other components. The AutoDock 1.5.7 software was used to convert the key target proteins and active ingredients from pdb format to pdbqt format, and to identify the active binding pockets. Finally, molecular docking was performed using AutoDock Vina.

### 2.3 Statistical analysis

The data were analyzed using SPSS 26.0 statistical software, and graphs were plotted using GraphPad Prism 7.0 software. For measurement data, a normality test was first conducted. If the data conformed to a normal distribution, they were expressed as mean ± standard deviation (Mean ± SD). A *t*-test was used for comparisons between two groups, and for comparisons among multiple groups, the least significant difference (LSD) method in one-way analysis of variance (ANOVA) was used when the variances were homogeneous, or Tamhane’s T2 was used when the variances were heterogeneous. If the data did not conform to a normal distribution, they were expressed as median (inter-quartile range) M (P25, P75). A rank-sum test was used for comparisons between two groups and among multiple groups. For count data, the chi-square test or Fisher’s exact probability test was used. When *p* < 0.05, it was considered statistically significant.

## 3 Results

### 3.1 The bioactive components and targets related to chronic cough in QFXFD

**Figure 1:**
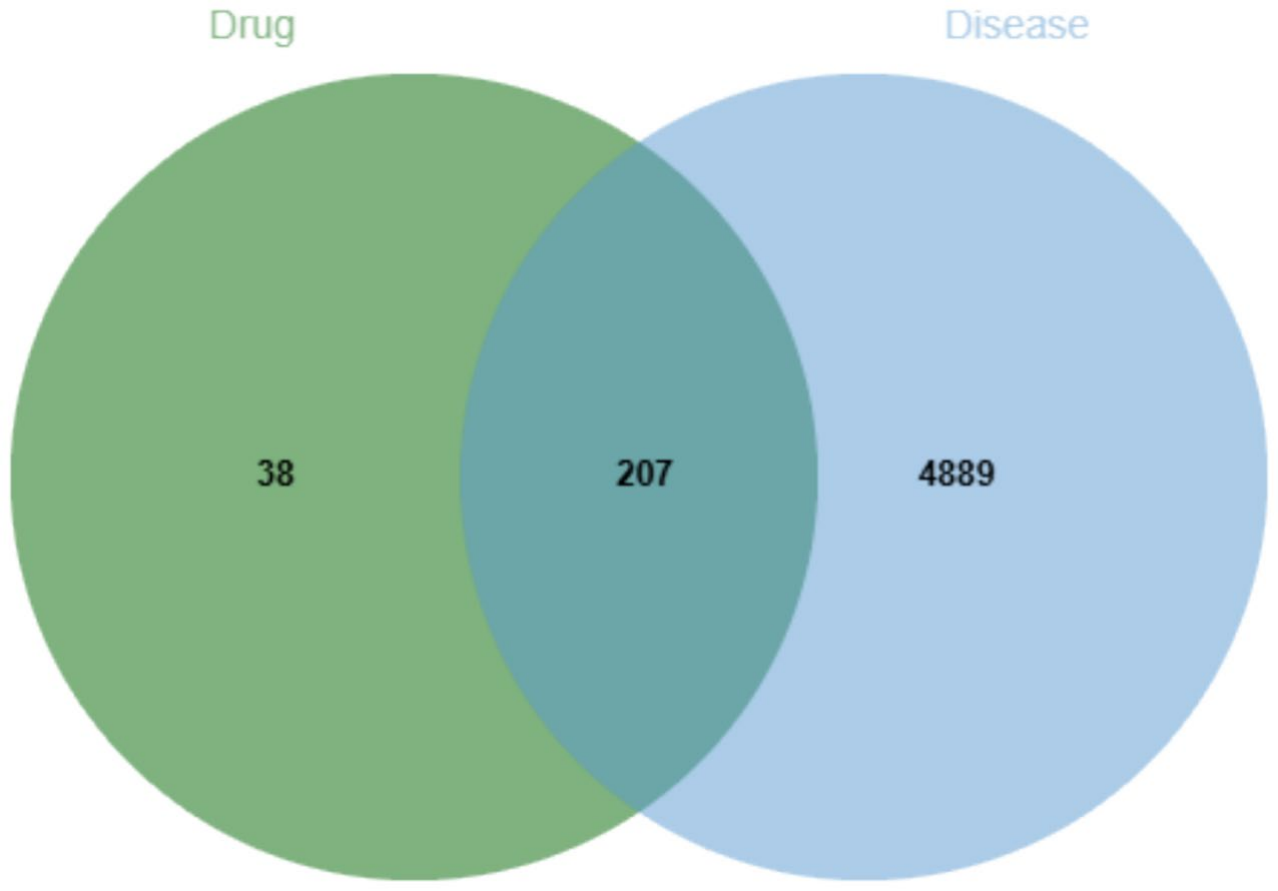
Venn diagram of the targets of QFXFD bioactive components and the primary relevant targets of chronic cough.

A total of 190 bioactive compounds from the constituent herbs of QFXFD were retrieved and screened from the TCMSP. Specifically, 32 compounds were identified from *Baibu*, 2 from *Houpo*, 23 from *Mahuang*, 24 from *Qianhu*, 6 from *Qingfengteng*, and 19 from *Ziwan*. The corresponding protein targets of these compounds were normalized and annotated using the UniProt database, with the filtering criteria set to “Popular organisms-Human” and “Status-Reviewed,” resulting in 1416 targets. After removing duplicates, 245 unique targets were retained. To identify disease-related targets, the keyword “chronic cough” was used to search the GeneCards, OMIM, and TTD databases, yielding 5,083, 54, and 1 target, respectively. After merging and deduplicating the datasets, a total of 5,096 unique disease-related targets were obtained. The intersection of these targets with those from the QFXFD resulted in 207 common target genes shared between the “drug” and “disease.” A network about QFXFD was built using Cytoscape 3.10.0 (Figure 1).

### 3.2 Construction of the “TCM-bioactive components-intersection targets” network

**Figure 2:**
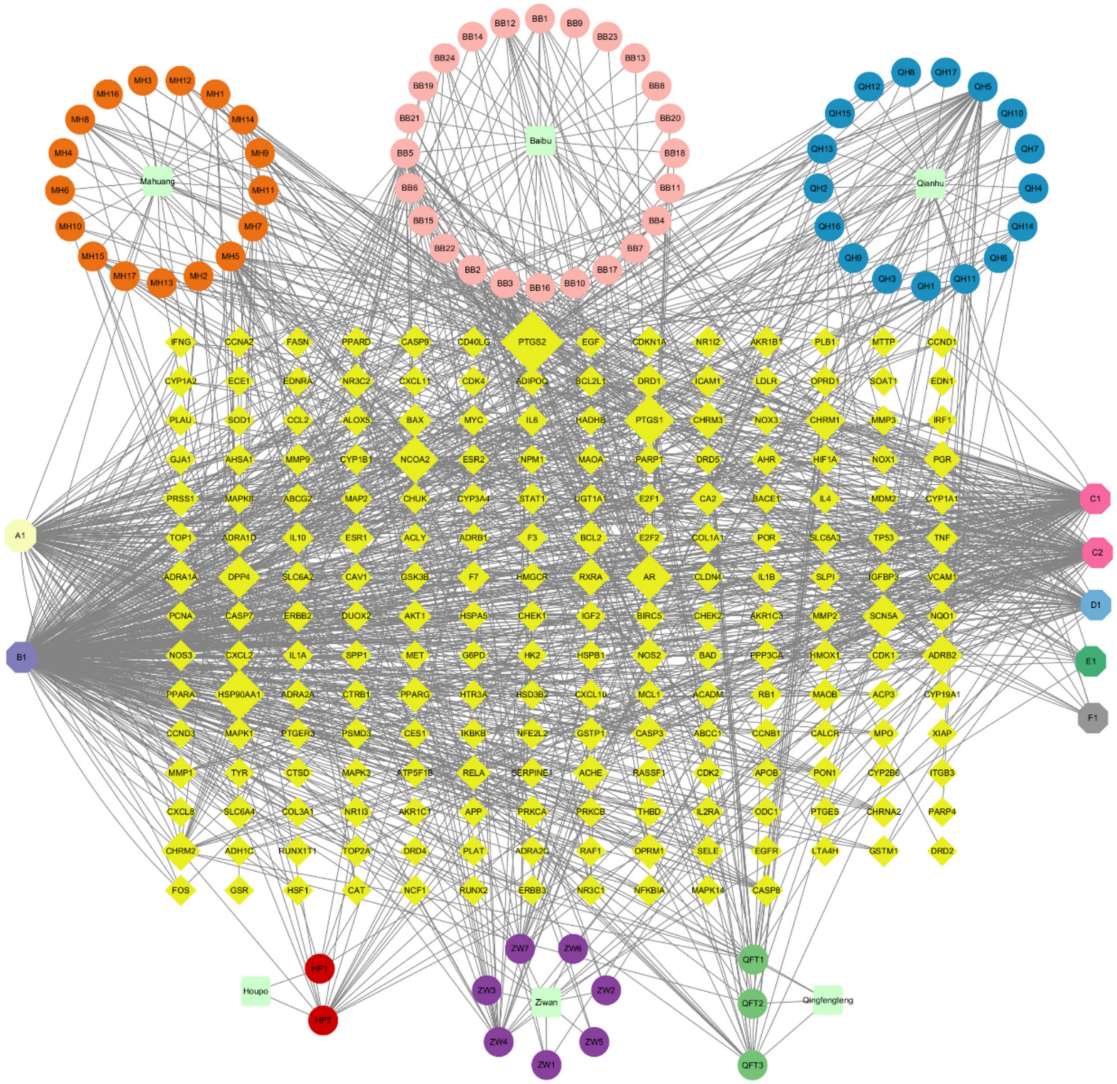
QFXFD network of relationships between the bioactive components and targets.

The “TCM-bioactive components-intersection targets “network was constructed using Cytoscape 3.10.0. This network consists of 290 nodes and 1,293 edges. The top 10 core components ranked by degree value are presented (Table 2). The top 6 active ingredients with the highest number of corresponding targets are quercetin, *β*-sitosterol, luteolin, kaempferol, stigmasterol, and tanshinone IIA, which may be the key components of the QFXFD for the treatment of chronic cough. A network about QFXFD was built using Cytoscape 3.10.0 (Figure 2). The color orange represents the active ingredients of *Mahuang*; pink represents the active ingredients of *Baibu*; blue represents the active ingredients of *Qianhu*; red represents the active ingredients of *Houpo*; purple represents the active ingredients of *Ziwan*; green represents the active ingredients of *Qingfengteng*; and yellow represents the targets. The larger the degree value, the larger the node shape.

**Table 2:** Core ingredients of QFXFD for the treatment of chronic cough.

Mol ID	Molecule name	Degree	OB (%)	DL	TCM
MOL000098	quercetin	363	46.43	0.28	*Mahuang, Qianhu, Ziwan*
MOL000358	beta-sitosterol	120	36.91	0.75	*Baibu, Mahuang, Qianhu, Qingfengteng, Ziwan*
MOL000006	luteolin	98	36.16	0.25	*Mahuang, Ziwan*
MOL000422	kaempferol	92	41.88	0.24	*Mahuang, Ziwan*
MOL000449	Stigmasterol	48	43.83	0.76	*Baibu, Mahuang*
MOL007154	tanshinone IIA	36	49.89	0.40	*Qianhu*
MOL004328	naringenin	31	59.29	0.21	*Mahuang*
MOL009374	7-methoxy-3-methyl-2,5-dihydroxy-9,10-dihydrophenanthrene	29	59.00	0.21	*Baibu*
MOL000392	formononetin	27	69.67	0.21	*Baibu,*
MOL000354	isorhamnetin	24	49.6	0.31	*Ziwan*

### 3.3 Analysis of a PPI network

**Figure 3:**
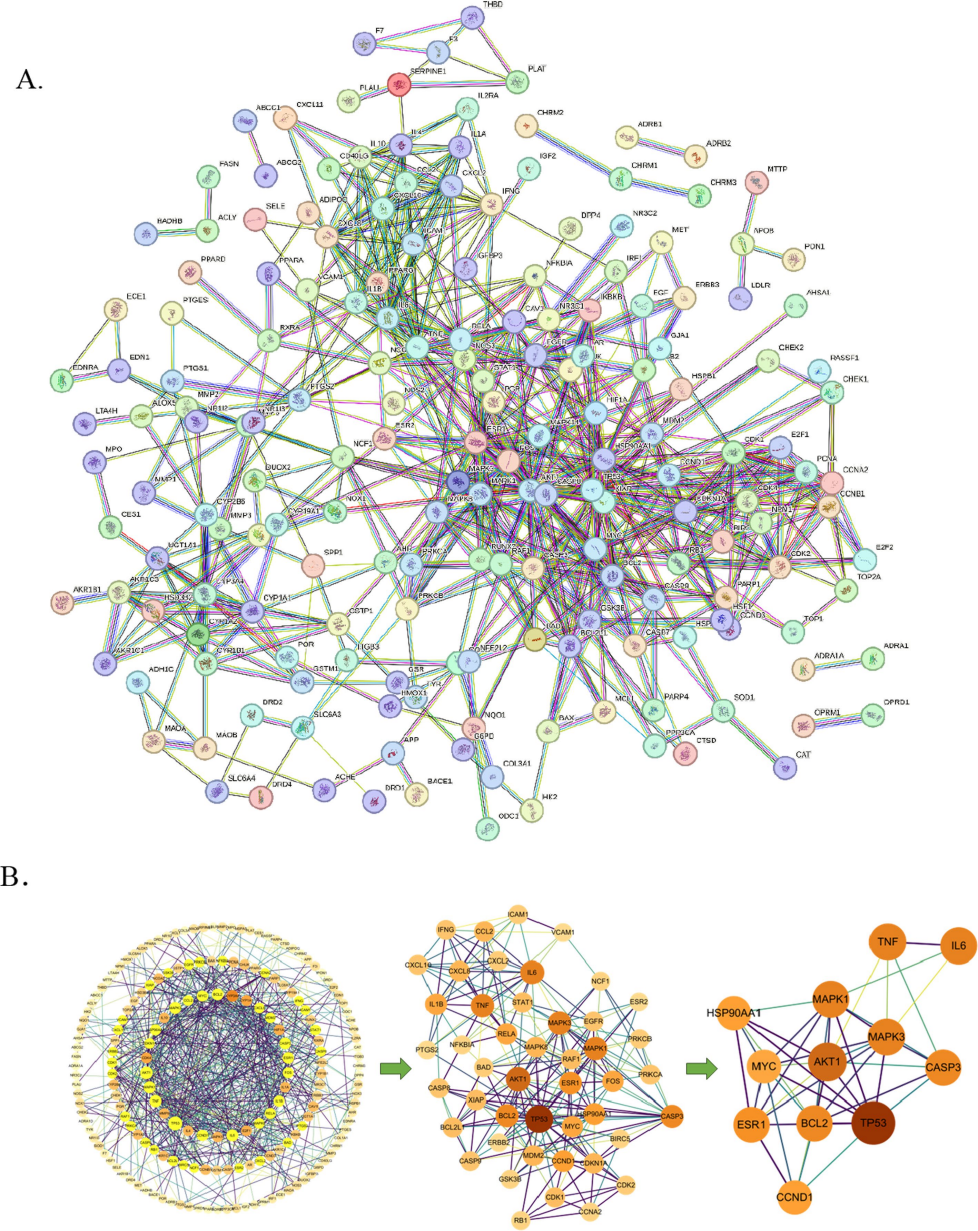
PPI network analysis of potential therapeutic targets for QFXFD intervention in chronic cough. **(A)** PPI network of intersection targets between QFXFD and chronic cough. **(B)** The core target screening process.

The 207 potential targets of QFXFD for the treatment of chronic cough were imported into STRING 12.0 to generate a PPI network. The network comprises 184 nodes and 621 edges, with an average node degree of 5.59. The CtyoNCA identified the top 12 hub genes, namely TP53, AKT1, IL6, MAPK1, MAPK3, TNF, BCL2, CASP3, ESR1, CCND1, HSP90AA1, MYC (Figure 3). The top 12 targets were shown in Table 3, ranked by degree. These targets may represent the core targets of QFXFD for the treatment of chronic cough.

**Table 3:** Information on core targets.

Targets	Degree	Betweenness	Closeness
TP53	27.0	422.2957871	0.671641791
AKT1	20.0	180.2635815	0.642857143
TNF	17.0	213.6909017	0.576923077
MAPK1	17.0	100.9214321	0.616438356
IL6	17.0	158.2680271	0.542168675
MAPK3	17.0	119.6998858	0.616438356
BCL2	16.0	53.81006267	0.555555556
CASP3	16.0	108.3067408	0.608108108
ESR1	15.0	62.93046743	0.584415584
CCND1	13.0	33.46711288	0.523255814
HSP90AA1	13.0	35.4209519	0.529411765
MYC	12.0	21.47795137	0.523255814

### 3.4 GO and KEGG analyses

**Figure 4:**
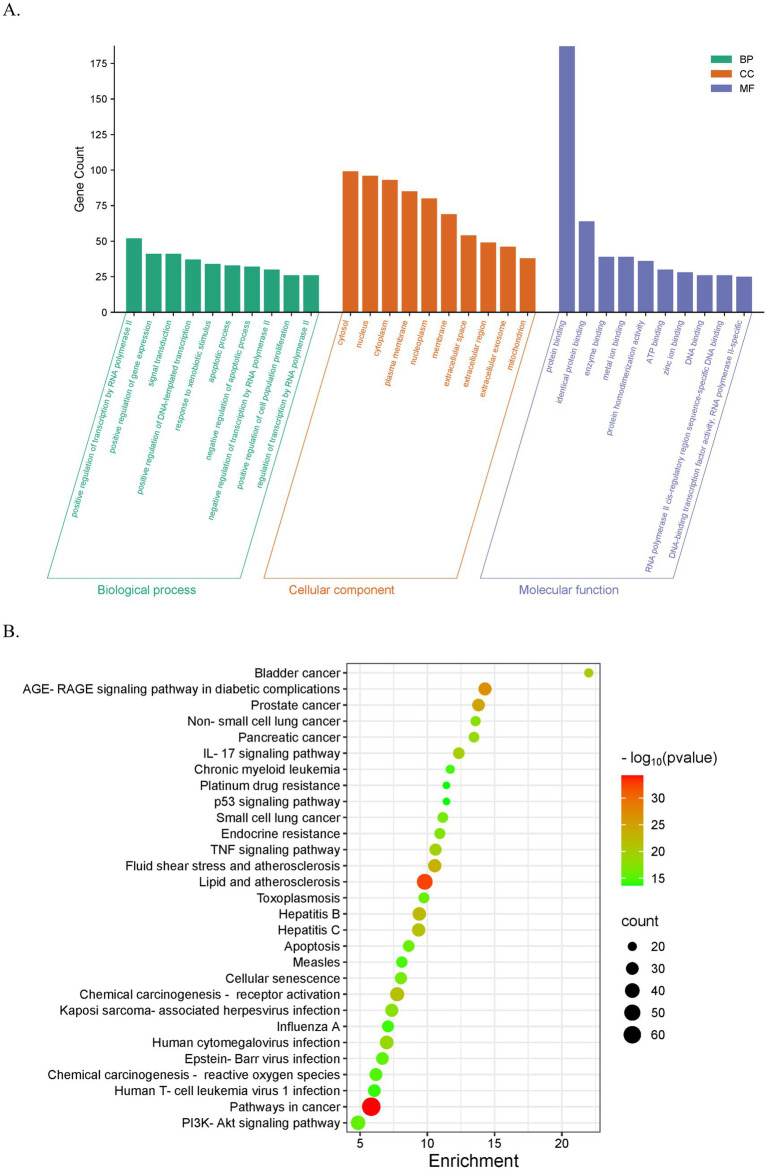
GO and KEGG analyses of potential therapeutic targets for QFXFD intervention in chronic cough. **(A)** The top 10 enriched entries for each GO category (BP, CC, and MF). **(B)** Top 30 results for KEGG analysis of 207 intersecting targets.

GO enrichment analysis identified a total of 838 BP, 104 CC, and 188 MF. The BP terms were primarily associated with RNA polymerase II-mediated transcription, gene expression, signal transduction, DNA-templated transcription, responses to exogenous stimuli, apoptosis, and regulation of cell population proliferation. The CC terms were mainly enriched in the cytosol, nucleus, cytoplasm, cell membrane, and mitochondria. The MF terms predominantly involved binding activities—such as protein–protein, enzyme, metal ion, ATP, and DNA binding—as well as specific biological activities including protein dimerization and DNA-binding transcription factor activity (Figure 4A).

KEGG pathway enrichment analysis was performed on the intersecting targets, with a significance threshold set at *p* < 0.05. A total of 179 pathways were identified, and the top 30 pathways were selected based on ascending *p* values. Among these, several pathways were closely associated with the pathogenesis of chronic cough, including the AGE-RAGE signaling pathway in diabetic complications, pathways in cancer, the IL-17 signaling pathway, TNF signaling pathway, PI3K-Akt signaling pathway, and the p53 signaling pathway (Figure 4B).

### 3.5 Molecular docking

**Figure 5:**
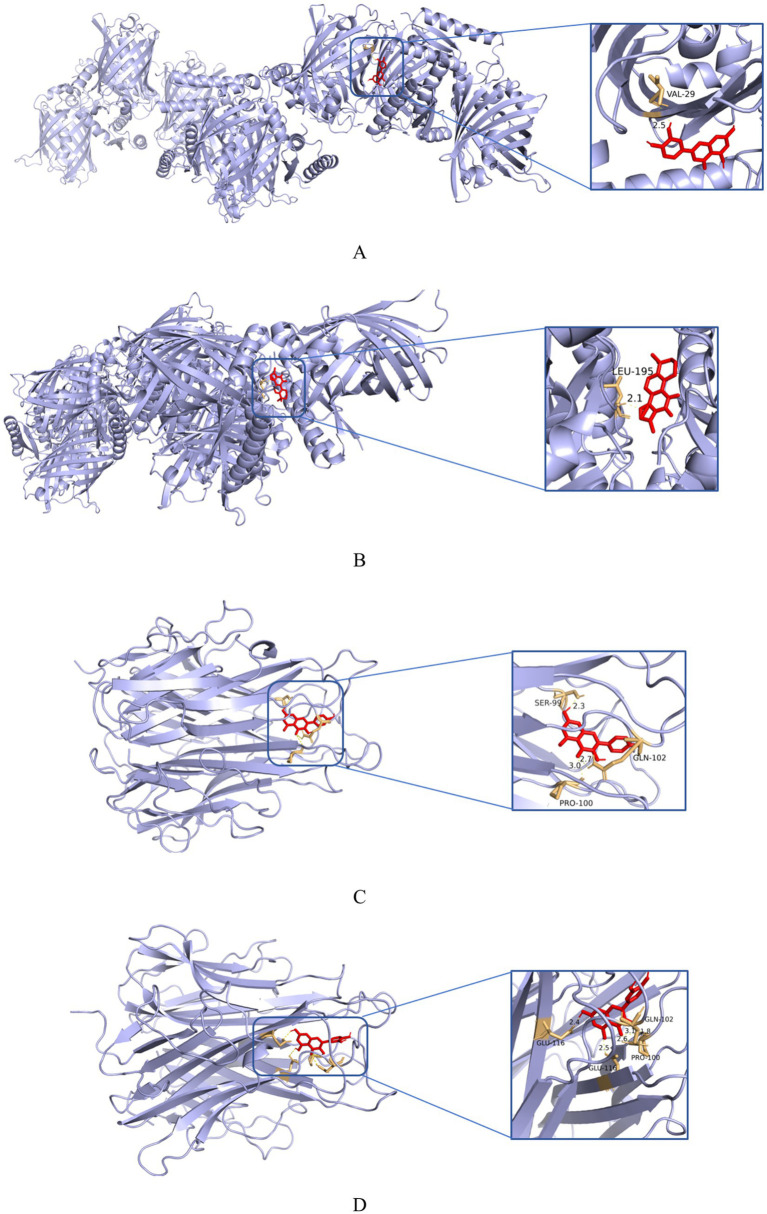
The outcomes of molecular docking between the core ingredients and targets: **(A)** Molecular docking results of luteolin and TP53(−9.1); **(B)** molecular docking results of tanshinone IIA and TP53(−9.6); **(C)** molecular docking results of kaempferol and TNF (−9.2); **(D)** molecular docking results of quercetin and TNF (−9.2).

TP53, AKT1, TNF, MAPK1, IL6 and MAPK3 were chosen as core targets ranked by Degree value. The six core ingredients (quercetin, beta-sitosterol, luteolin, kaempferol, stigmasterol and tanshinone II A) obtained from the previous analysis were molecularly docking with these six core targets (Table 4). A binding energy less than −4.25 kcal·mol^−1^ indicates a certain binding activity between the ligand and the receptor, less than −5.0 kcal·mol^−1^ suggests a better binding activity, and less than −7.0 kcal·mol^−1^ indicates a strong binding activity (23). As shown in Table 3, the binding energies between the core targets and the corresponding active components are all less than −5.0 kcal·mol^−1^, suggesting a better binding activity. The docking binding energies that performed the best were luteolin-TP53 (−9.1; Figure 5A), tanshinone IIA-TP53 (−9.6; Figure 5B), kaempferol-TNF (−9.2; Figure 5C), quercetin-TNF (−9.2; Figure 5D).

**Table 4:** Information on molecular docking binding energy.

Core targets	Core ingredients	TCM	kcal·mol^−1^
TP53	quercetin	*Mahuang, Qianhu, Ziwan*	−8.9
	luteolin	*Mahuang, Ziwan*	−9.1
	tanshinone IIA	*Qianhu*	−9.6
AKT1	kaempferol	*Mahuang, Ziwan*	−6.9
	quercetin	*Mahuang, Qianhu, Ziwan*	−6.8
	luteolin	*Mahuang, Ziwan*	−6.7
	naringenin	*Mahuang*	−6.3
TNF	kaempferol	*Mahuang, Ziwan*	−9.2
	quercetin	*Mahuang, Qianhu, Ziwan*	−9.2
	luteolin	*Mahuang, Ziwan*	−8.9
	dl-praeruptorin a	*Qianhu*	−6.3
MAPK1	quercetin	*Mahuang, Qianhu, Ziwan*	−8.4
	luteolin	*Mahuang, Ziwan*	−8.6
	naringenin	*Mahuang*	−6.7
IL6	quercetin	*Mahuang, Qianhu, Ziwan*	−6.7
	luteolin	*Mahuang, Ziwan*	−7.1
MAPK3	naringenin	*Mahuang*	−7.0

### 3.6 Results of the verification in animal experiments

#### 3.6.1 General condition of guinea pigs

During the feeding period, guinea pigs in all groups exhibited good general health, with normal activity, appropriate food intake, and glossy fur. During the modeling process, two guinea pigs in the model group, and one each in the TRPA1 inhibitor group and TRPV1 inhibitor group, died. Following successful modeling, most guinea pigs displayed signs of accelerated and deepened respiration along with frequent abdominal muscle contractions. A few exhibited loud, distinctive coughs, accompanied by symptoms such as hair loss, reduced food intake, and decreased activity. By the time of cough frequency assessment, except for the normal control group, each remaining group had eight surviving guinea pigs.

#### 3.6.2 QFXFD reduced the cough hypersensitivity of guinea pigs

**Figure 6:**
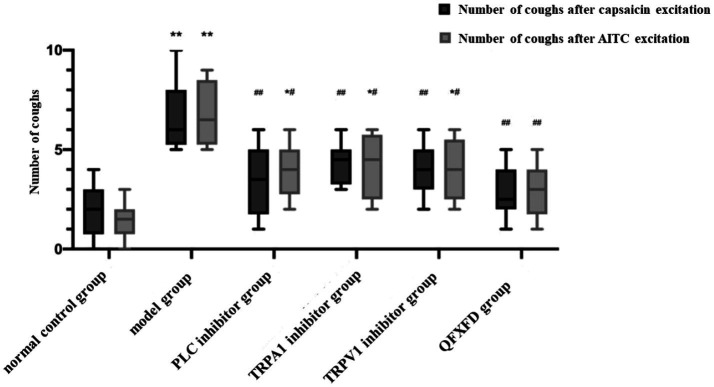
The cough reflex sensitivity (CRS) of guinea pigs. ^*^p < 0.05, ^**^p < 0.01, vs. normal control group; ^#^*p* < 0.05, ^##^*p* < 0.01, vs. model group.

Cough measurement showed that, compared to the normal control group, the total cough frequency of guinea pigs in the model group significantly increased (*p* < 0.01). Compared with the model group, the total cough frequency of guinea pigs in each treatment group showed a significant decrease (*p* < 0.01 or *p* < 0.05). There was no significant difference (*p* > 0.05) between the Western medicine treatment groups and the traditional Chinese medicine group. Furthermore, there were no significant differences (*p* > 0.05) observed in the cough frequency following stimulation with capsaicin or AITC among the different groups (Figure 6).

#### 3.6.3 Effect of QFXFD on leukocyte classification and count in BALF of guinea pigs

Compared with the normal control group, the total leukocyte count in the BALF of the model group was significantly increased (*p* < 0.01), primarily due to elevated levels of neutrophils and eosinophils (*p* < 0.01).

**Figure 7:**
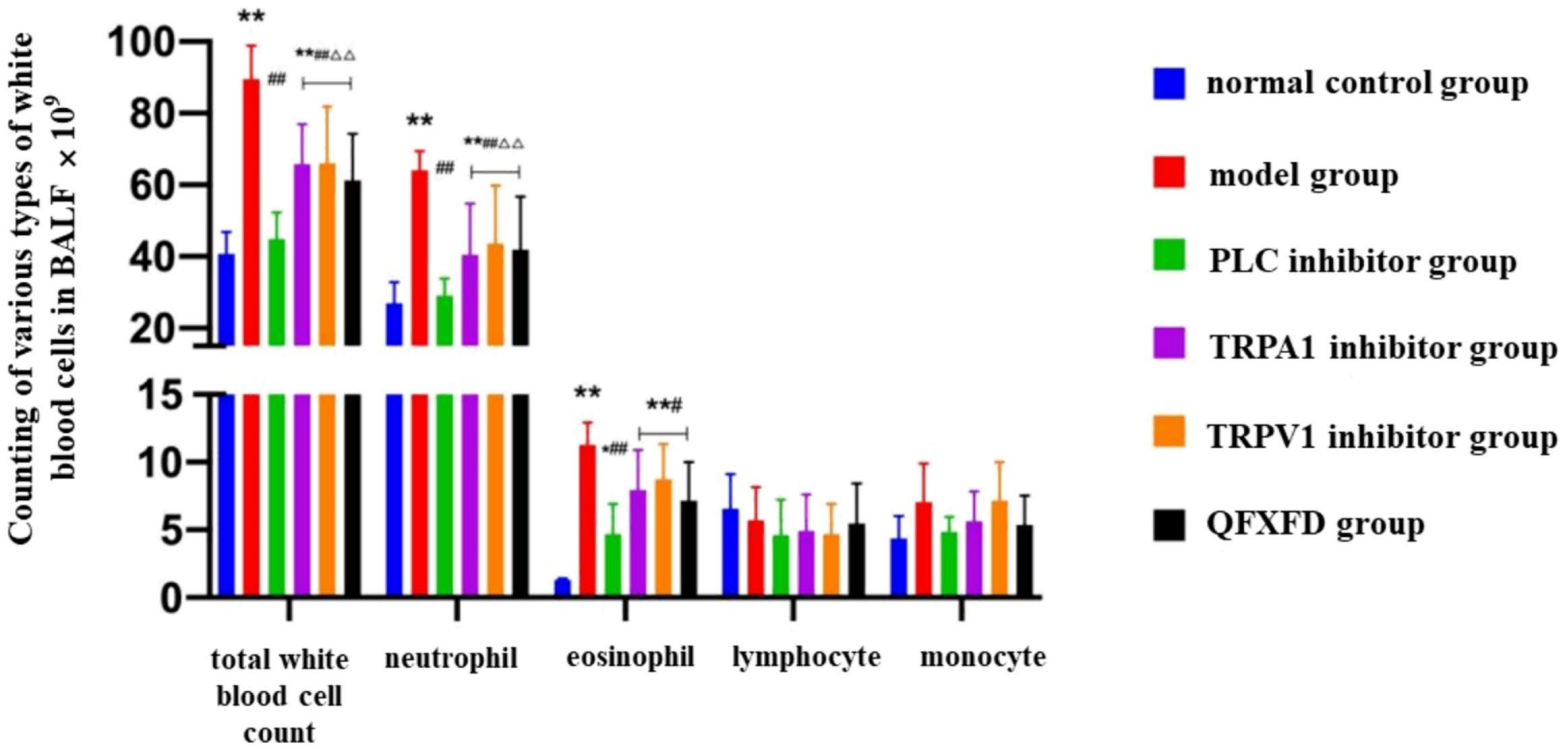
Differential leukocyte classification and counts in BALF across experimental groups. ^*^p < 0.05, ^**^p < 0.01, vs. normal control group; #*p* < 0.05, ^##^*p* < 0.01, vs. model group; ^ΔΔ^*p* < 0.01, vs. PLC inhibitor group.

In comparison with the model group, all treatment groups—including the Western medicine groups and the QFXFD group—exhibited a significant reduction in total leukocyte count, mainly attributed to decreased neutrophil and eosinophil levels (*p* < 0.05, *p* < 0.01). However, when compared with the PLC inhibitor group, the TRPA1 inhibitor group, TRPV1 inhibitor group, and QFXFD group showed significantly higher total leukocyte and neutrophil counts (*p* < 0.01), while the increase in eosinophils was not statistically significant (*p* > 0.05). There were no significant differences in lymphocyte or monocyte counts among the groups (*p* > 0.05; Figure 7).

#### 3.6.4 Effect of QFXFD on the pathological changes of lung tissues in guinea pigs

The alveolar structure in the normal control group was intact, with no inflammatory cell infiltration. In the model group, the alveolar walls were thickened, with a large number of inflammatory cells infiltrating the tissue, blood cell exudation, narrowed bronchial lumen, and local bronchial wall damage, including epithelial cell shedding. These findings indicate that the cough hypersensitivity model was successfully established.

**Figure 8:**
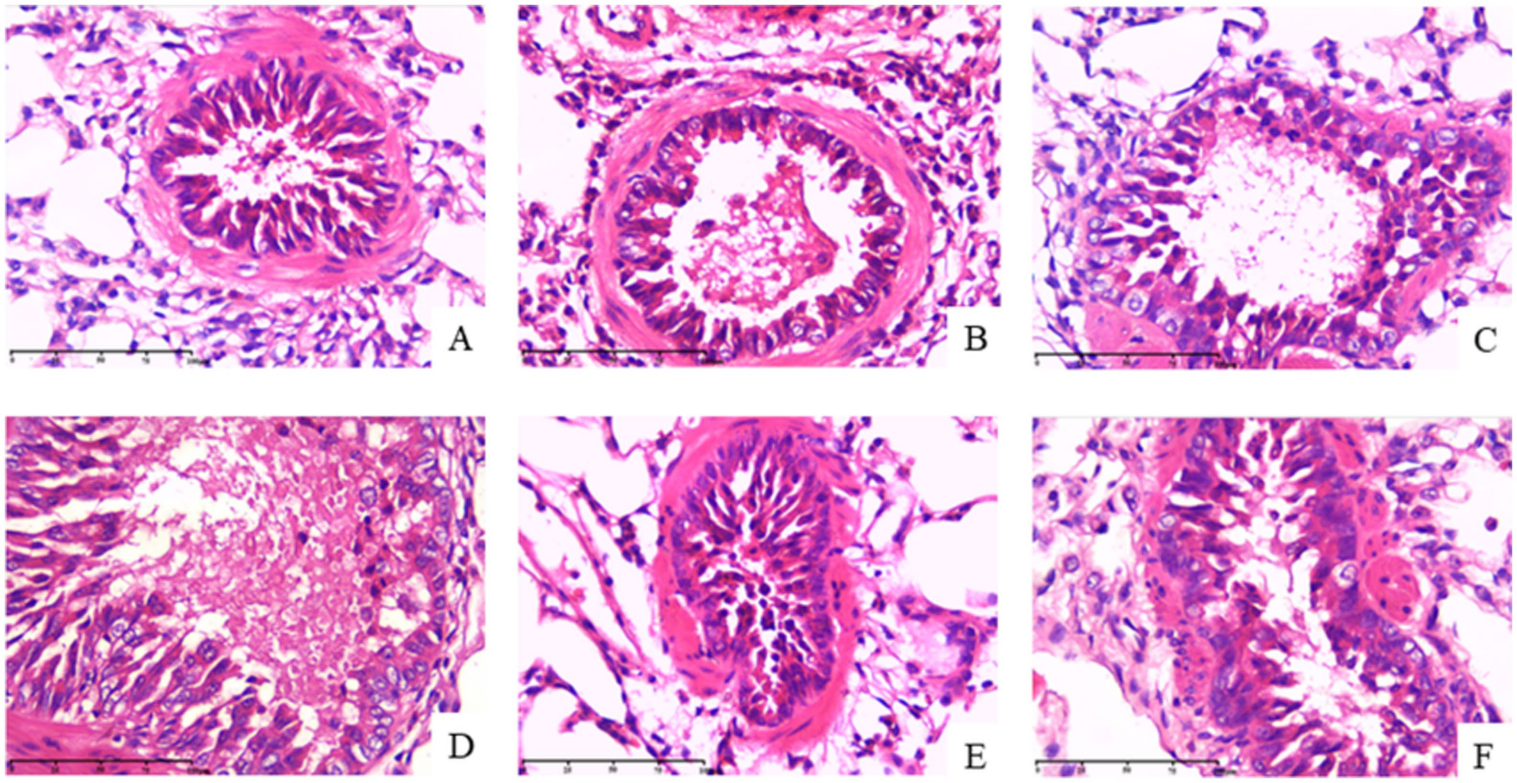
QFXFD attenuated the inflammatory injury of lung tissue. Representative images of HE-stained (×400 magnification). **(A)** Normal control group; **(B)** model group; **(C)** PLC inhibitor group; **(D)** TRPA1 inhibitor group; **(E)** TRPV1 inhibitor group; **(F)** QFXFD group.

Compared with the model group, the lesions in the PLC inhibitor group and the wind-dispelling and lung-ventilating formula group were milder, with only a few or localized inflammatory cell infiltrations. In the TRPA1 inhibitor group and TRPV1 inhibitor group, the alveolar walls were slightly thickened, with localized minor damage and thickened bronchial walls. However, the bronchial lumen remained relatively normal, with only a few inflammatory cells infiltrating locally. Overall, the TRPA1 inhibitor group, TRPV1 inhibitor group, PLC inhibitor group, and wind-dispelling and lung-ventilating formula group all showed improvements compared with the model group (Figure 8).

#### 3.6.5 Effect of QFXFD on PLC-*β*/PKC, TRPA1, TRPV1, NK-1R protein expression in lung tissue

Western blot analysis of PLC-β and PKC protein expression in guinea pig lung tissues revealed that, compared to the normal control group, all groups except the PLC inhibitor group exhibited significantly elevated protein levels (*p* < 0.01), while the PLC inhibitor group showed no significant difference (*p* > 0.05). Compared to the model group, protein expression in the PLC inhibitor group, TRPA1 inhibitor group, TRPV1 inhibitor group, and QFXFD group was significantly reduced (*p* < 0.01). Among these, the PLC inhibitor group exhibited markedly lower protein expression than the TRPA1 inhibitor group, TRPV1 inhibitor group, and QFXFD group (*p* < 0.01), while no significant differences were observed among the latter three groups (*p* > 0.05).

**Figure 9:**
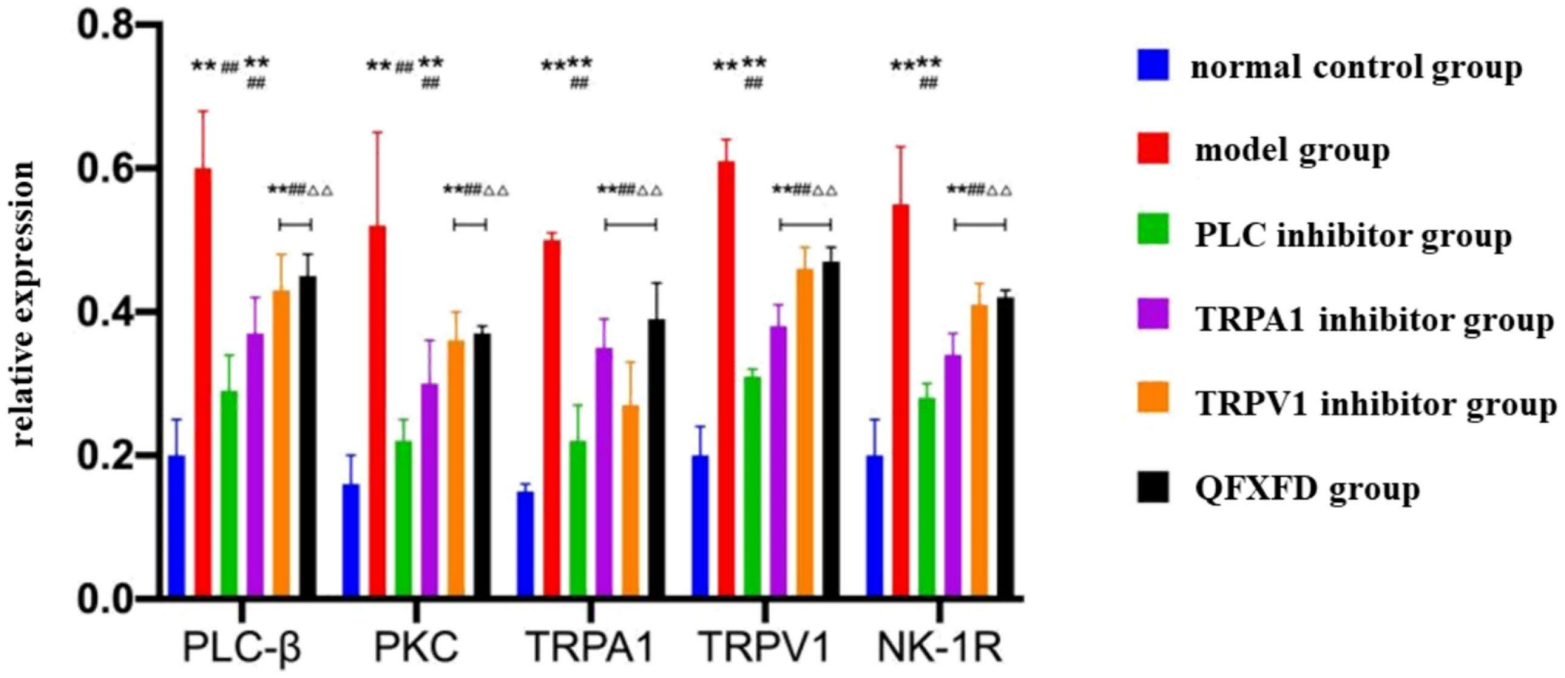
Effect of QFXFD on PLC-*β*/PKC, TRPA1, TRPV1, NK-1R protein expression in lung tissue. ^**^p < 0.01, vs. normal control group; ^##^*p* < 0.01, vs. model group; ^ΔΔ^*p* < 0.01, vs. PLC inhibitor group.

**Figure 10:**
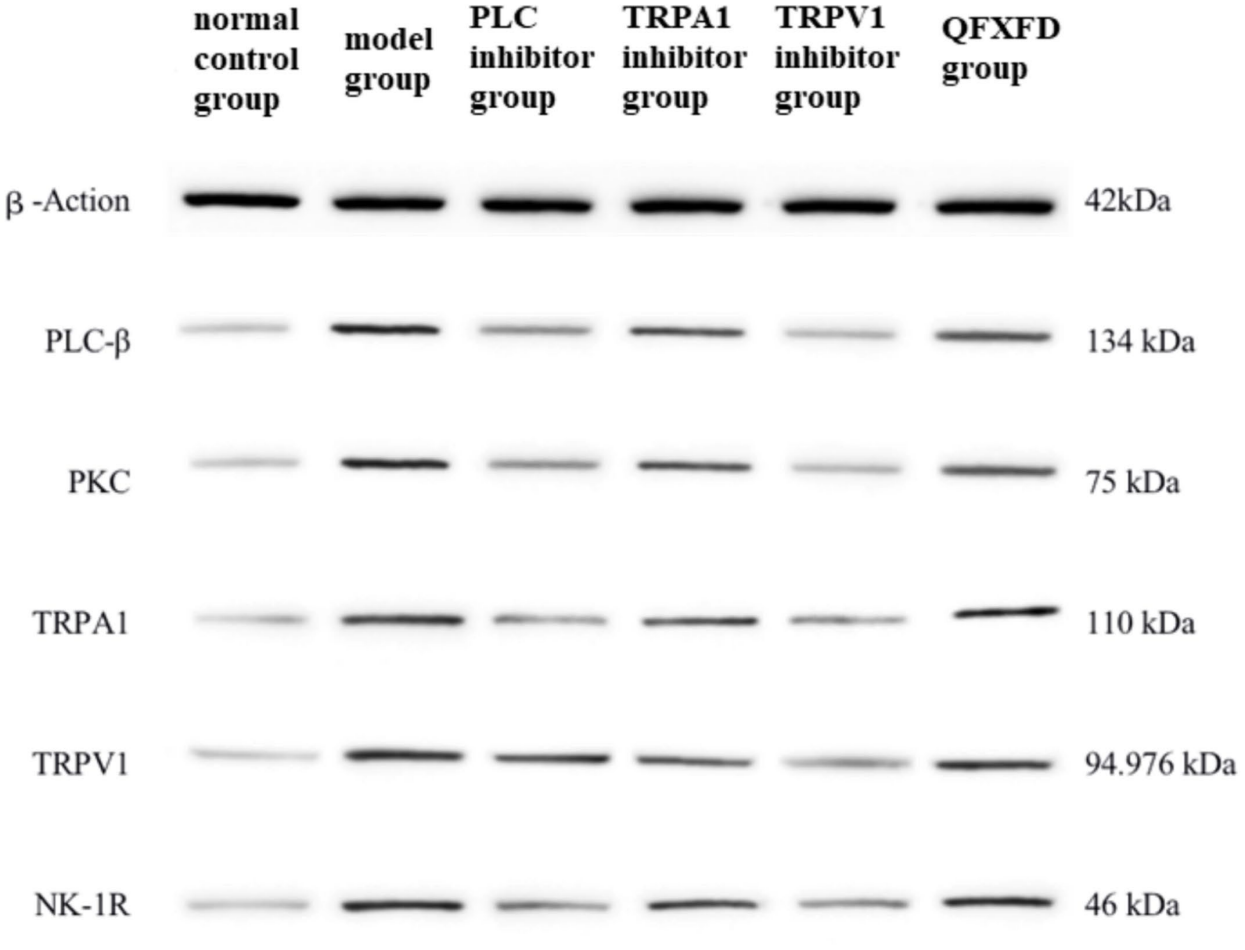
The protein blots and quantitative analysis of PLC-β, PKC, TRPA1, TRPV1, NK-1R in the lung tissue.

Similarly, Western blot analysis of TRPV1, TRPA1, and NK-1R protein expression demonstrated significantly increased expression in all groups compared to the normal control group (*p* < 0.01). Compared to the model group, the PLC inhibitor group, TRPA1 inhibitor group, TRPV1 inhibitor group, and QFXFD group all showed significantly decreased protein expression (*p* < 0.01). Again, the PLC inhibitor group displayed significantly lower expression levels than the TRPA1 inhibitor group, TRPV1 inhibitor group, and QFXFD group (*p* < 0.01), with no significant differences among the latter three groups (*p* > 0.05; Figures 9, 10).

#### 3.6.6 Effect of QFXFD on PLC-*β*/PKC, TRPA1, TRPV1, SP mRNA expression in lung tissue

**Figure 11:**
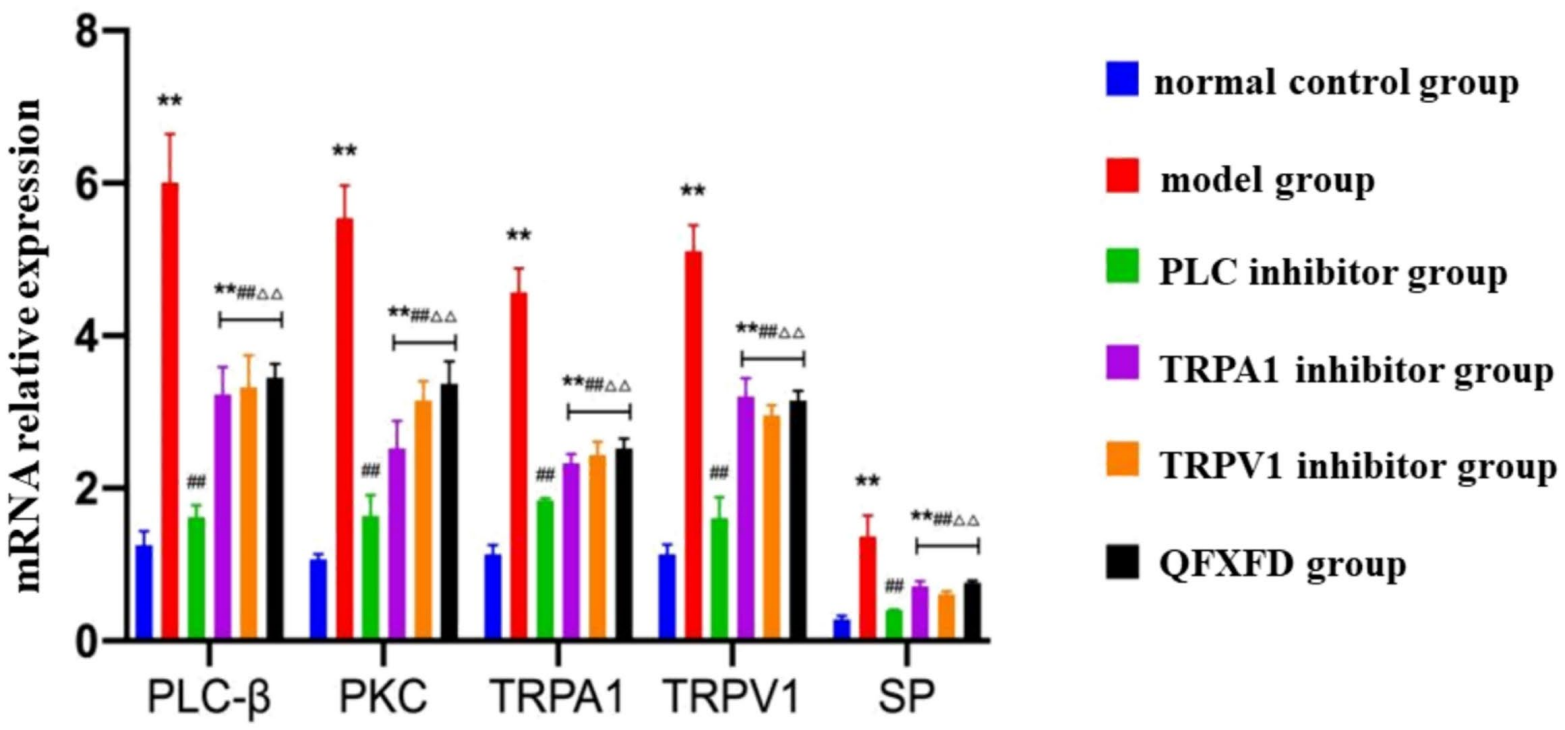
Effect of QFXFD on PLC-β/PKC, TRPA1, TRPV1, SP mRNA expression in lung tissue. ^**^p < 0.01, vs. normal control group; ^##^*p* < 0.01, vs. model group; ^ΔΔ^*p* < 0.01, vs. PLC inhibitor group.

RT-PCR analysis of mRNA expression levels of PLC-β, PKC, TRPA1, TRPV1, and SP revealed that, compared with the normal control group, all groups except the PLC inhibitor group exhibited significantly increased mRNA expression (*p* < 0.01). Compared with the model group, mRNA expression levels in the PLC inhibitor group, TRPA1 inhibitor group, TRPV1 inhibitor group, and QFXFD group were significantly reduced (*p* < 0.01). The PLC inhibitor group showed a significantly greater reduction in mRNA expression compared to the TRPA1 inhibitor group, TRPV1 inhibitor group, and the QFXFD group (*p* < 0.01). No significant differences in mRNA expression were observed among the TRPA1 inhibitor group, TRPV1 inhibitor group, and QFXFD group (*p* > 0.05; Figure 11).

## 4 Discussion

Chronic cough falls under the categories of “persistent cough” and “stubborn cough” in TCM. The fundamental pathogenesis is “wind pathogen invading the lung”. The TCM characteristic of “Wind” being “active and rapidly changing” (characterized by its paroxysmal and migratory nature) aligns closely with the modern medical concepts of airway hyperresponsiveness and hypersensitivity of sensory nerve endings. Recent studies have further proposed that neurogenic inflammatory neuropeptides and related inflammatory cytokines may serve as the biological material basis for this “wind pathogen invading the lung” (24). In the prescription for QFXFD, the chief herb, *Mahuang*, dispels wind, disperses pathogenic factors, and promotes the flow of lung qi. *Qianhu* helps to expel wind pathogens, clear heat, and transform phlegm. It works in synergy with *Mahuang*, with one herb dispersing and the other descending, ensuring that lung qi is both promoted and regulated. *Houpo* has the ability to descend qi and resolve phlegm, assisting *Qianhu* in regulating lung qi, while also descending the qi of the large intestine, harmonizing both lung and large intestine. *Baibu* and *Ziwan* are used to stop cough and transform phlegm. *Qingfengteng* dispels wind and dispels dampness, enhancing the wind-dispelling power when combined with *Mahuang* and *Qianhu*. This prescription has shown good clinical efficacy, and previous animal and cellular studies have initially revealed the pharmacological mechanisms of this formula. It has been shown to reduce cough frequency in guinea pigs, decrease airway hyperresponsiveness, and alleviate neurogenic inflammation in the airways (16, 25–29). Our experimental results demonstrate that the formula significantly inhibits the PLC/PKC-TRPA1/V1 pathway, which essentially reveals the modern biological essence of “wind-dispelling” (*Qufeng*) herbs. At the molecular level, these findings validate that”wind-dispelling” (*Qufeng*) can effectively suppress cough hypersensitivity, thereby providing a modern scientific rationale for the TCM clinical strategy of treating chronic cough from the perspective of “Wind.”

### 4.1 Network pharmacology

Given the complexity and diversity of active ingredients and potential targets of QFXFD in the body, we employed network pharmacology to analyze its main components. The 10 core components identified include quercetin, *β*-sitosterol, luteolin, kaempferol, stigmasterol, tanshinone IIA, and hesperetin, among others. Quercetin is a common flavonoid compound with natural antioxidant activity. It participates in oxidative stress reactions in the body, limits inflammatory responses, inhibits the release of pro-inflammatory factors, and also alleviates smooth muscle spasms, improving airway hyperreactivity (30). A study exploring the P2X3 inhibitor gefapixant as a substitute for refractory chronic cough found that quercetin could inhibit P2X3 activation by blocking IP-HD conformational changes, reducing cough frequency without causing dysgeusia (31). Stigmasterol and β-sitosterol are plant sterols with similar pharmacological effects, including anti-inflammatory, antioxidant, and immune-regulating actions. They exert mucosal protective effects by enhancing SOD activity and inhibiting the release and aggregation of inflammatory factors (32). Research indicates that β-sitosterol can improve asthma symptoms in mice and reduce airway inflammation (33). Stigmasterol exerts a dose-dependent anti-inflammatory and antioxidant stress effect and reduces NK1-R expression induced by IL-13 in BEAS-2B cells (34). Stigmasterol also alleviates airway inflammation in OVA-induced asthma mice by inhibiting the TGF-β1/Smad2 and IL-17A signaling pathways (35). Luteolin has anti-inflammatory, anti-allergic, and immune-enhancing effects. It can regulate bronchial smooth muscle contraction and reduce airway hyperreactivity (36), lower serum IgE levels, reduce IL-4 and IL-5 levels in BALF, and increase IFN-*γ* expression. Kaempferol has broad pharmacological effects, including anti-inflammatory, antioxidant, anti-tumor, and immune-modulatory actions. Research suggests that its anti-inflammatory mechanism primarily involves inhibiting the TLR4/MyD88-mediated NF-κB pathway (37). Hesperetin, a naturally occurring flavonoid, has extensive pharmacological activity in respiratory diseases and can play a role in treating allergic asthma by exerting anti-inflammatory, antioxidant, and airway remodeling effects (38, 39). Tanshinone IIA, one of the most pharmacologically active compounds extracted from *Salvia miltiorrhiza*, exhibits anti-inflammatory, antioxidant, anti-atherosclerotic, and anti-tumor properties. Tanshinone IIA reduces eosinophils in allergic asthma mice, alleviates lung inflammation and mucin production, and decreases IL-4 and IL-13 expression in bronchoalveolar lavage fluid and lung tissue. It also improves airway inflammation and hyperreactivity in asthma mice (40, 41). Tanshinone IIA sodium sulfate, a water-soluble derivative of tanshinone IIA, relaxes smooth muscle in the mouse trachea (42).

The PPI network analysis revealed that the effective components of QFXFD may improve chronic cough-associated airway neurogenic inflammation through targets such as TP53, AKT1, IL6, MAPK1, MAPK3, TNF, BCL2, CASP3, ESR1, CCND1, HSP90AA1, and MYC. Among the core targets, TP53 is a well-known tumor suppressor, and its mutation in normal airway epithelium is associated with an increased risk of lung cancer (43). However, emerging evidence indicates that it also plays a critical role in regulating pulmonary redox homeostasis and inflammatory damage (44). Furthermore, TP53 can maintain airway epithelial integrity by regulating the expression of tight junction proteins (45). AKT1, a subtype of AKT, is an important downstream target of the PI3K-Akt signaling pathway. AKT1 exerts its biological effects by activating PI3K and binding to its downstream targets. The PI3K/Akt pathway is a critical pathological process in airway hyperreactivity (46). The extracellular signals that initiate cellular responses are transmitted by MAPKs, which regulate a variety of cellular activities. MAPK1 and MAPK3 belong to the MAPK family. Most respiratory diseases are associated with dysregulation of the MAPK signaling pathway, and MAPK is considered an important potential therapeutic target (47). Research indicates that MAPK is involved in airway hyperreactivity (48), airway inflammation (49), and airway remodeling (50, 51), and it mainly functions in signal pathways interacting with various substances. TNF and IL-6 are critical cytokines that play a key role in inflammation and immune responses. They often work synergistically in the respiratory system, participating in inflammatory reactions and disease progression. Both TNF and IL-6 play important roles in the development of airway inflammation in most respiratory diseases. In the pathogenesis of bronchial asthma, IL-6 and TNF serve as key intermediates in signal transduction and also play significant roles in improving airway hyperreactivity and alleviating airway inflammation (52, 53). IL-6 and TNF-*α* are also considered major pro-inflammatory factors strongly associated with sepsis (54).

The results of GO functional enrichment analysis suggest that the treatment of chronic cough with the QFXFD primarily involves biological processes such as RNA polymerase II-mediated transcription, gene expression, signal transduction, DNA template transcription, response to external stimuli, apoptosis, and cell proliferation. The biological components include the cytoplasm, nucleus, cytosol, cell membrane, mitochondria, etc. Molecular functions include protein–protein interactions, enzyme activity, binding with metal ions, ATP, DNA, and protein dimerization, as well as DNA-binding transcription factor activity. These findings indicate that the core components of the formula may exert their effects through these enriched pathways. From the KEGG pathway enrichment analysis, it is inferred that this process may be closely related to cancer-related pathways, lipid metabolism and atherosclerosis, AGE-RAGE signaling in diabetic complications, fluid shear stress and atherosclerosis, chemical carcinogen-receptor activation, IL-17 signaling pathway, TNF signaling pathway, PI3K/Akt signaling pathway, p53 signaling pathway, and others. The PI3K/Akt signaling pathway plays a significant role in respiratory diseases, closely related to the regulation of inflammatory cells, inflammatory signaling, immune cell responses, autophagy, oxidative stress, and airway remodeling (55–57). The PI3K/Akt signaling pathway regulates smooth muscle contraction and relaxation, release of inflammatory mediators, and airway neuroregulation, enhancing excessive airway responsiveness to stimuli. It is a critical pathophysiological process in airway hyperresponsiveness (46). Over-activation of the TNF signaling pathway is associated with various chronic inflammatory diseases. As a key pro-inflammatory cytokine, TNF-*α* participates in the progression of chronic cough by driving airway inflammation, neurosensitization, and tissue remodeling (58). Similarly, the abnormal activation of the IL-17 signaling pathway is linked to chronic cough pathology. IL-17 can suppress the function of Treg cells or promote Th17 differentiation, leading to an imbalance in the Th17/Treg ratio, which maintains a persistent inflammatory state and prolongs the disease course (59). Furthermore, IL-17 is closely related to airway inflammation, with Th17-mediated immune responses playing a critical role in the pathogenesis of airway inflammation in asthma (60). In conclusion, the molecular docking results suggest that the core components of QFXFD have a good binding affinity with protein targets, with higher docking scores and tighter chemical bonds. This preliminary evidence supports the reliability of the network pharmacology approach discussed in this study and indirectly suggests that the core components of QFXFD have an improving effect on chronic cough.

Notably, there is a clear functional intersection between the broad signaling networks identified via network pharmacology and the specific PLC/PKC-TRPA1/V1 axis validated in our animal experiments. Research has demonstrated that the PI3K/Akt signaling pathway acts as a pivotal regulatory node that can directly upregulate the expression and activity of its downstream effector protein, PKC, thereby modulating airway function (61). Meanwhile, the inflammatory milieu driven by the TNF and IL-17 signaling pathways serves as a catalyst for airway neurogenic inflammation; these pro-inflammatory cytokines significantly enhance the sensitization and membrane expression of TRPA1/V1 channels, effectively lowering the cough threshold (62). Consequently, QFXFD likely attenuates the sensitization of the PLC/PKC-TRPA1/V1 axis by modulating these global inflammatory networks. This logical bridge, transitioning from holistic network regulation to specific molecular intervention, provides robust mechanistic support for the therapeutic efficacy of QFXFD in treating chronic cough.

### 4.2 Animal experiments

PLC is an enzyme that plays a crucial role in intracellular signaling pathways, and there are several isoforms of PLC, with PLC-*β* being one of the important subtypes (63). The main function of PLC is to catalyze the breakdown of phosphatidylinositol-(4,5)-bisphosphate (PIP2) into inositol-triphosphate (IP3) and diacylglycerol (DAG), which are 2 s messenger molecules (64). IP3 and DAG play significant roles in various signaling pathways. IP3 can activate intracellular Ca^2+^ channels, leading to an increase in Ca^2+^ concentration, while DAG can activate PKC, further regulating intracellular signaling pathways. PKC is a key protein kinase in cellular signal transduction, and its activation is lipid-dependent and Ca^2+^-dependent. It requires the presence of membrane lipids, such as DAG, and an increase in cytosolic Ca^2+^ concentration. The PLC-PKC process can influence the opening of cation channels and activate voltage-dependent, non-selective cation channels—transient receptor potential channels (TRP) (65). TRPA1 and TRPV1 are well-studied members of the TRP channel family, which are widely expressed in the airways and lungs. Studies have confirmed that TRPA1 and TRPV1 interact with each other, showing a channel-linked relationship. TRPA1/V1 can be activated by various endogenous and exogenous substances, producing neuropeptide mediators that induce neurogenic inflammation and result in cough hypersensitivity (66). When PLC is activated by endogenous or exogenous mediators, it causes PIP2 hydrolysis, leading to the generation of IP3 and DAG. This, in turn, activates PKC, triggering a cascade reaction that opens the TRPA1/V1 channels. DAG is an endogenous activator of TRPV1, directly activating the TRPV1 pathway in a membrane-segregated manner. Studies have shown that PKC is involved in the activation of the TRPV1 pathway by DAG (67). IP3 induces the release of intracellular Ca^2+^, which, combined with the Ca^2+^ influx caused by the opening of the TRPV1 channel, can also activate the TRPA1 pathway. Thus, a joint sensitization and channel interaction between the PLC/PKC-TRPA1/V1 pathways is formed.

After the activation of TRPA1/V1, it induces the release of neuropeptides such as substance P (SP), calcitonin gene-related peptide (CGRP), and neurokinin A (NKA) from C fibers. These neuropeptides can act on corresponding receptors on effector cells, leading to increased microvascular permeability, plasma extravasation, and bronchoconstriction, as well as the activation of inflammatory cells and the release of inflammatory mediators. This, in turn, promotes the occurrence and development of airway inflammation, resulting in neurogenic airway inflammation (68). SP and other neuroinflammatory mediators, upon binding to their corresponding receptors on effector cells, can reactivate the PLC/PKC pathway, triggering a cascade reaction and sensitizing TRPA1/V1 (69). This forms a positive feedback signal transduction mechanism, leading to the sustained presence of neurogenic airway inflammation and the persistence of chronic cough symptoms. The above process outlines the mechanism: “Endogenous and exogenous substances → TRPA1/V1 activation → Activation of the PLC/PKC pathway triggers Ca^2+^ influx in sensory neurons→Neuropeptide production → Airway neurogenic inflammation.”

Based on the above mechanism, we observed the expression levels of PLC-*β*, PKC, TRPA1, TRPV1, and SP/NK-1R in lung tissue from each group of animals to investigate the synergistic action of the TRPA1 and TRPV1 channels, the upstream PLC/PKC regulatory mechanism, and the effect of this pathway on airway-originated inflammation and cough sensitivity. The aim was to explore the pathological mechanism of increased cough sensitivity and the pharmacodynamic mechanisms of both traditional Chinese and Western medicines. The experimental results showed that after 7 days of drug intervention, the general condition and cough frequency of guinea pigs in the Western medicine treatment groups and the QFXFD group improved compared to the model group. Eosinophils and neutrophils are important factors in triggering airway inflammation and causing airway hyperresponsiveness (70). The PLC inhibitor, TRPA1 inhibitor, TRPV1 inhibitor, and QFXFD all significantly reduced the extent of airway inflammation, mainly by decreasing neutrophils and eosinophils. Cell classification counts from bronchoalveolar lavage fluid and lung tissue pathology indicated that the QFXFD group was more effective in alleviating airway inflammation. Among all the treatment groups, RT-PCR and Western blot results both suggested that the PLC inhibitor could reduce the expression levels of PLC-β, PKC, TRPA1, TRPV1, and NK-1R proteins, as well as the mRNA expression levels of PLC-β, PKC, TRPA1, TRPV1, and SP. The PLC inhibitor significantly suppressed the expression of various indicators in the pathway and had a good alleviating effect on neurogenic airway inflammation. The QFXFD showed a slightly weaker improvement in these indicators compared to the PLC inhibitor but was comparable to the TRPA1 and TRPV1 inhibitors, with no clear advantage or disadvantage. In conclusion, the QFXFD reduced cough frequency in the guinea pig model, alleviated airway inflammation, and effectively suppressed the expression of PLC, PKC, TRPA1, TRPV1, and SP/NK-1R at various levels. Its mechanism of action may be related to the regulation of the PLC/PKC-TRPA1/V1 pathway, alleviating neurogenic airway inflammation and thus reducing cough sensitivity.

## 5 Conclusion

This study, based on network pharmacology, has preliminarily predicted the active components, drug targets, and drug-disease related pathways of QFXFD. The findings suggest that QFXFD improves chronic cough through a multi-target and multi-pathway regulatory mechanism, primarily involving the modulation of inflammatory responses, immune responses, and other bodily functions. Animal experiments also validated that QFXFD has a certain effect on improving cough sensitivity and airway neurogenic inflammation. These results provide a foundation for further research on the prevention and treatment of chronic cough with QFXFD, offering a modern scientific rationale for the TCM strategy of treating cough from the perspective of “Wind.”

However, this study has certain limitations: the final sample size was reduced to 8 per group due to severe allergic responses during the modeling process, and direct functional validations for specific targets were not conducted. Future research will focus on larger-scale cohorts and in-depth molecular experiments to further substantiate these findings. In terms of clinical application, QFXFD has demonstrated favorable efficacy in numerous previous studies. However, the extrapolation of findings from animal models to clinical practice still lacks high-quality clinical evidence. Future research should involve multi-center, randomized, double-blind, and placebo-controlled trials. Furthermore, standardized assessment tools should be employed to evaluate cough hypersensitivity and rigorously assess the clinical therapeutic potential of QFXFD.

## Data Availability

The original contributions presented in the study are included in the article/Supplementary material, further inquiries can be directed to the corresponding authors.

## Footnotes

1
https://old.tcmsp-e.com/tcmsp.php

2
https://www.genecards.org/

3
https://omim.org/

4
https://db.idrblab.net/ttd/

5
https://david.ncifcrf.gov/

6
https://www.bioinformatics.com.cn/

7
http://www.rcsb.org

8
https://pubchem.ncbi.nlm.nih.gov
