# Correction: Investigation of the active compounds and pharmacological mechanisms of *Qufengxuanfei decoction* in the treatment of chronic cough

**DOI:** 10.3389/fmed.2026.1876231

**Published:** 2026-05-21

**Authors:** Zhilin Liu, Jiashan Li, Kun Ji, Shaohui Wen, Shangjuan Dong, Ying Wang, Jingyue Luo, Jianling Ma, Liqing Shi

**Affiliations:** 1 The First Affiliated Hospital of Guangzhou Medical University, Guangzhou, Guangdong, China; 2 The First Affiliated Hospital of Guangzhou Medical University, Hengqin Hospital/Hengqin Guangdong-Macao In-Depth Cooperation Zone Central Hospital, Hengqin, Guangdong, China; 3 Second School of Clinical Medicine, Beijing University of Chinese Medicine, Beijing, China; 4 Institute of Acupuncture and Moxibustion, China Academy of Chinese Medical Sciences, Beijing, China; 5 Department of Respiration, Dongfang Hospital, Beijing University of Chinese Medicine, Beijing, China; 6 Department of Respiration, Zhejiang Provincial Hospital of Traditional Chinese Medicine, Hangzhou, Zhejiang, China; 7 Department of Traditional Chinese Medicine, Beijing Chest Hospital, Capital Medical University, Beijing, China

**Keywords:** airway neurogenic inflammation, animal experiment, chronic cough, molecular docking, network pharmacology, Qufengxuanfei decoction

The Funder “The Beijing High-Level Innovation and Entrepreneurship Talent Support Program Young Top Talent Projects (G202524100) and The National Natural Science Foundation of China (No. 82474472)” to the authors was erroneously omitted.

The original version of this article has been updated.

