# Supplementary material for: Investigation of the active compounds and pharmacological mechanisms of Qufengxuanfei decoction in the treatment of chronic cough
Source: Front Med (Lausanne). 2026 Apr 30;13:1748916. doi: 10.3389/fmed.2026.1748916 (PMC13171490; doi:10.3389/fmed.2026.1748916)
Supplement: Supplementary file 1 [file Data_Sheet_1.docx]

**Supplementary Material: Chemical Characterization of QFXFD by UPLC-Q-TOF-MS**

**1. Instruments and Reagents**

The chemical profiling of the QFXFD decoction was performed using a SYNAPT G2 UPLC-Q-TOF-MS high-resolution mass spectrometer (Waters, USA). Other equipment included a BP211D precision analytical balance (Sartorius, Germany) and a KQ-800KDB ultrasonic cleaner (Kunshan, China). Formic acid and acetonitrile were of mass spectrometry grade (Fisher Scientific, USA). Distilled water was used throughout the study.

**2. Preparation of QFXFD**

QFXFD was prepared by the Pharmaceutical Department of Dongfang Hospital, Beijing University of Chinese Medicine. The raw herbs were extracted twice with 10 volumes of water for 1.5 hours each time. The extract was concentrated to a relative density of 1.05 at 60°C. Ethanol (95%) was added to reach a final concentration of 60%. After standing, the supernatant was collected, ethanol was recovered, and the solution was concentrated to a total volume of 6000 mL.

**3. Methods**

**Chromatographic Conditions:** Column: Waters ACQUITY UPLC BEH C_18_ (2.1mm×100mm, 1. 7μm) with a Van Guard Pre-Column (2.1mm×5mm，1.7μm). Mobile Phase: 0.1% formic acid in water (A) and 0.1% formic acid in acetonitrile (B). Gradient Elution: 0–15 min, 95%–60% A; 15.1–17.0 min, 5% A; 17.1–20.0 min, 95% A. Parameters: Column temperature: 40°C; Flow rate: 0.4 mL/min; Injection volume: 4μL.

**Mass Spectrometry Conditions:** Ionization: Electrospray ionization (ESI) in positive and negative modes. Voltages: Source voltage: 2700 V (positive) and 2100 V (negative); Cone voltage: 40 V. Temperatures: Source temperature: 120°C; Desolvation temperature: 500°C. Gas Flow: Desolvation gas flow rate: 800 L/h. Calibration: Leucine-enkephalin (LE) was used for mass calibration (m/z 556.2771 for positive mode, m/z 554.2615 for negative mode). Scan Range: TOF-MS scan range: 100–1500 Da. Sample Preparation: The decoction was centrifuged at 12,000 rpm for 10 min twice. A 100μL aliquot of the supernatant was dissolved in 900μL of 0.1% formic acid water to obtain the test solution.

**4. Data Analysis**

A compound database for QFXFD was established and imported into UNIFI software for peak extraction and automatic matching. Compound structures were confirmed by comparing chromatography and mass spectrometry data with reference standards, isotopic matching, and fragment information. Manual verification was performed based on literature-reported data.

**5. Results**

Experimental Results 1: The total ion chromatograms of UPLC-TOF-MS component identification under positive and negative ion scan modes are shown in Figure 1.


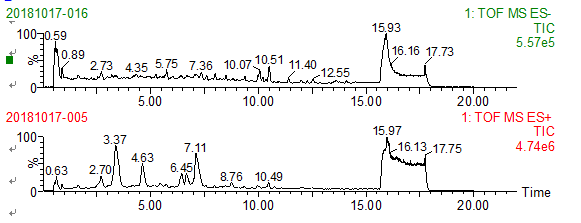


**Figure 1 Total ion chromatograms**

Experimental Results 2: Identification of Active Components in Qufeng Xuanfei Formula

**Table 1. Major components identified in QFXFD via UPLC-TOF-MS (Positive Ion Mode)**

| Retention Time (min) | Molecular Formula | Compound | TCM |
| --- | --- | --- | --- |
| 3.37 | C_19_H_23_NO_4_ | Sinomenine | *Qingfengteng* |
| 4.63 | C_20_H_24_NO_4_ | Magnoflorine | *Houpo* |
| 7.11 | C_22_H_33_NO_4_ | Tuberostemonine | *Baibu* |
| 8.63 | C_24_H_26_O_7_ | Praeruptorin B | *Qianhu* |
| 8.76 | C_22_H_33_NO_6_ | Oxytuberostemonine | *Baibu* |
| 10.34 | C_21_H_36_O_10_ | AShionoside A | *Ziwan* |
| 12.66 | C_10_H_15_NO | Ephedrine | *Mahuang* |

**Table 2. Major components identified in QFXFD via UPLC-TOF-MS (Negative Ion Mode)**

| Retention Time (min) | Molecular Formula | Compound | TCM |
| --- | --- | --- | --- |
| 8.5 | C_25_H_33_N_5_O_8_ | Astin A | *Ziwan* |
| 16.03 | C_18_H_18_O_2_ | Honokiol | *Houpo* |
| 16.41 | C_18_H_18_O_2_ | Magnolol | *Houpo* |
